# Supplementary figures and images for: Polymodal allosteric regulation of Type 1 Serine/Threonine Kinase Receptors via a conserved electrostatic lock
Source: PLoS Comput Biol. 2017 Aug 21;13(8):e1005711. doi: 10.1371/journal.pcbi.1005711 (PMC5578689; doi:10.1371/journal.pcbi.1005711)

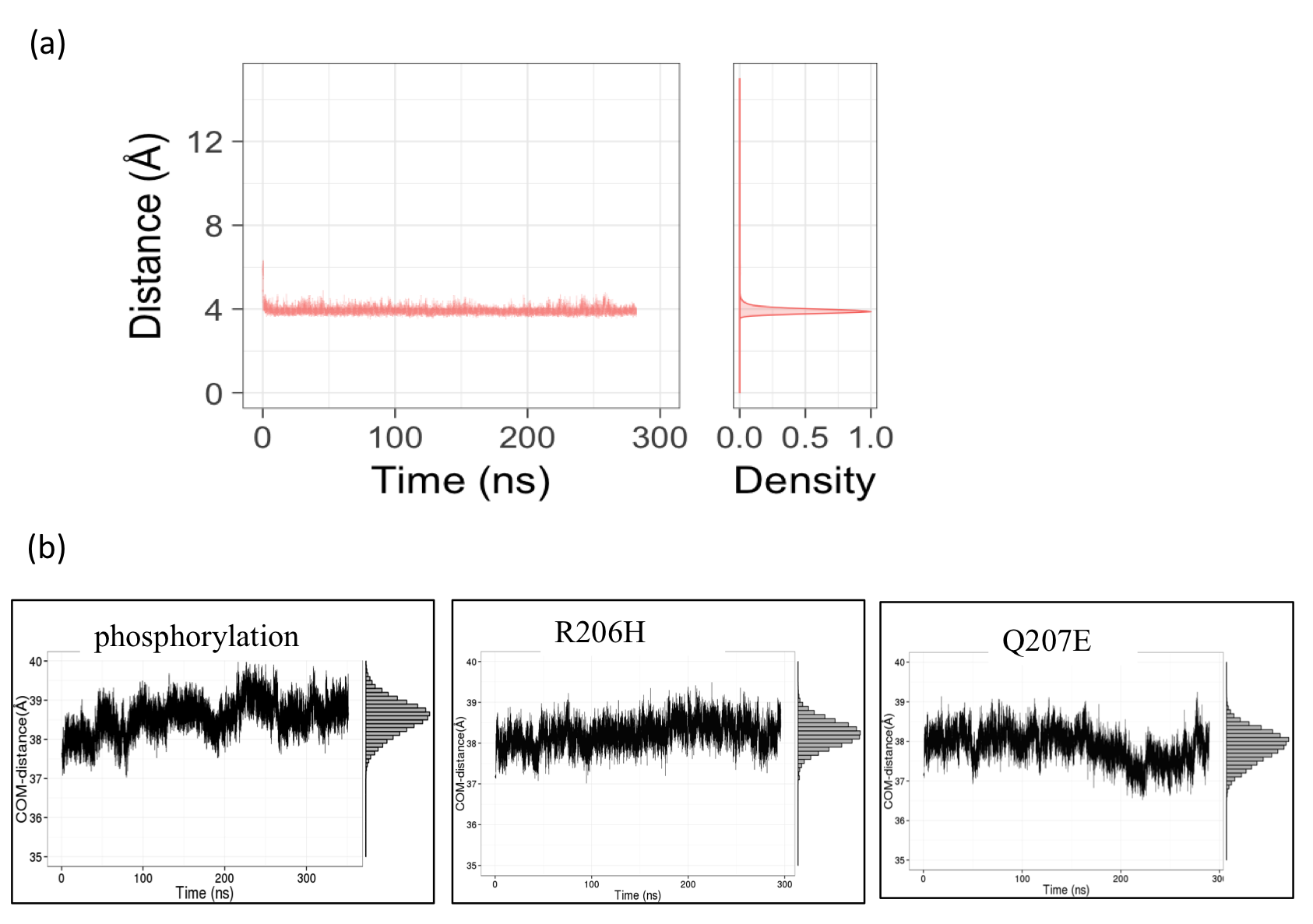

Supplement: S1 Fig — (a) Salt bridge distance between R375(Cζ) and D354 (Cγ) from a duplicated simulation of FKBP12-ALK2WT. (b). Center of mass distance between FKBP12 and ALK2 during 300 ns simulations of FKBP12-ALK2WT-Phosp, FKBP12-ALK2R206H, and FKBP12-ALK2Q207E. (TIF) [file pcbi.1005711.s001.tif]

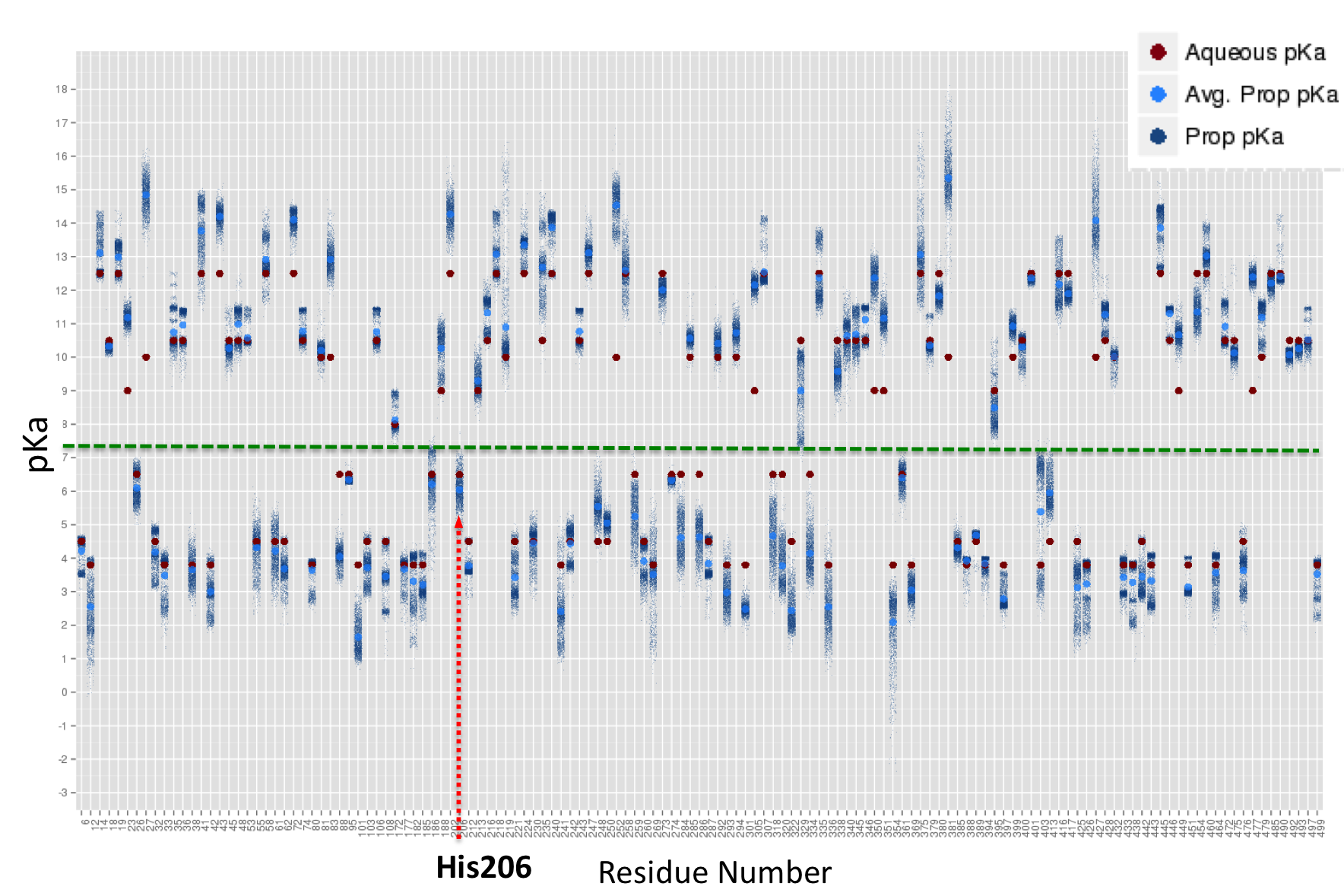

Supplement: S2 Fig — Dark blue dots represent the pKa value from each snapshot, light blue dots represent average value, and red dots represent pKa in aqueous solution. Green dashed line indicates physiological pH 7.4. His206 is indicated by red dashed arrow. (TIF) [file pcbi.1005711.s002.tif]

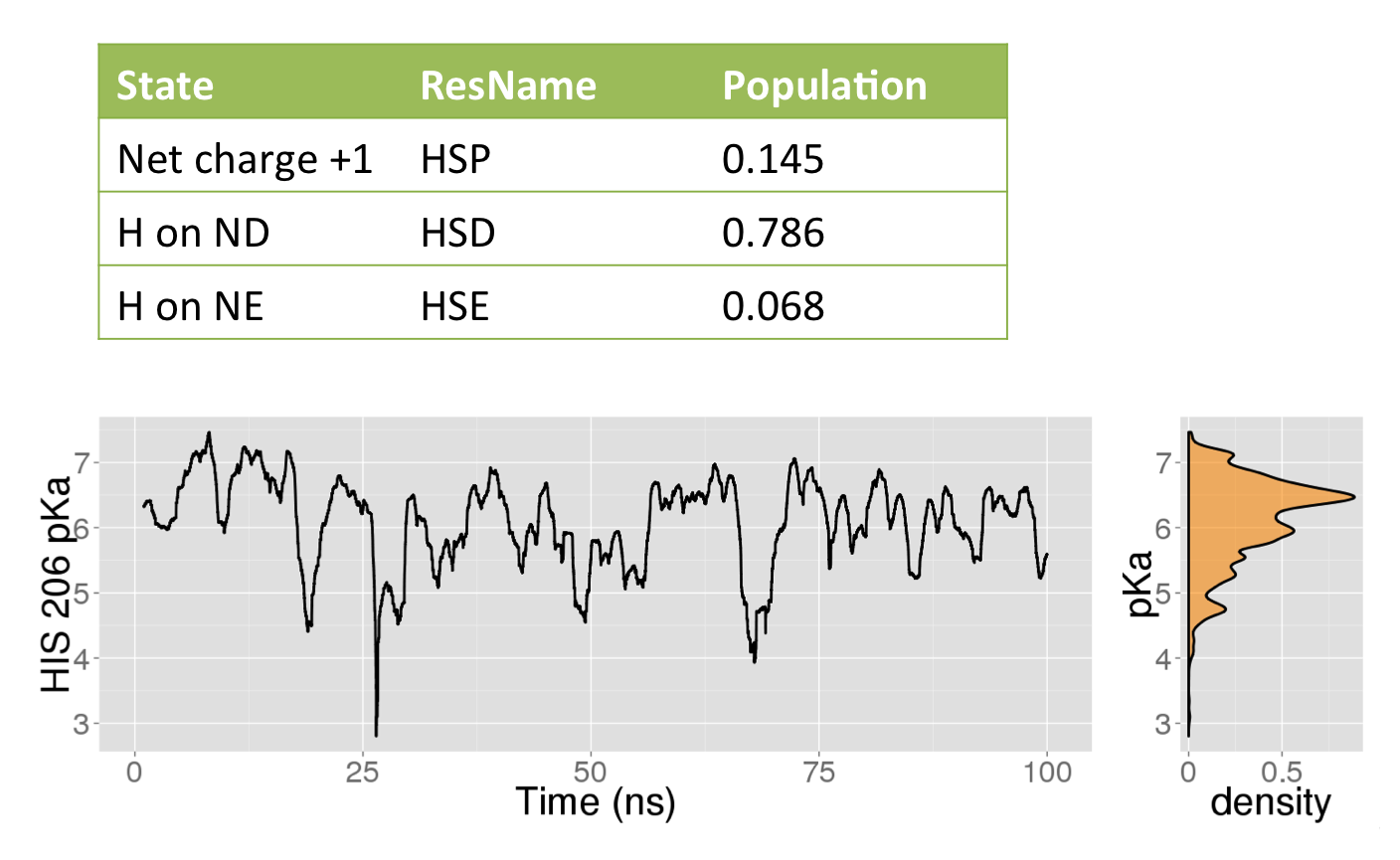

Supplement: S3 Fig — The population of His206 protonation state is shown in the top table. The calculated pKa of His206 is plotted against simulation time. Right: a density plot of calculated pKa of His206 during the simulation. (TIF) [file pcbi.1005711.s003.tif]

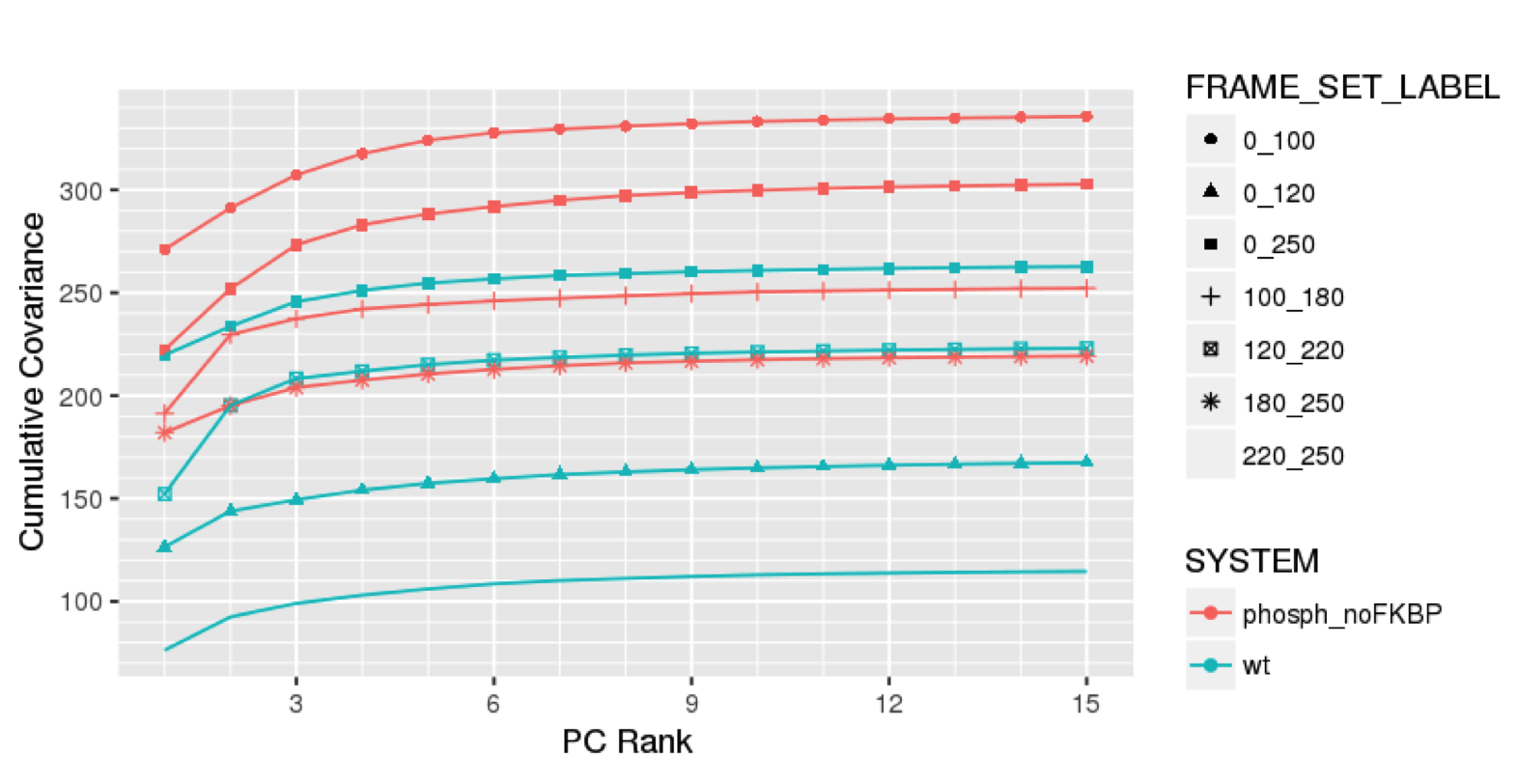

Supplement: S4 Fig — FKBP12-ALK2WT is shown in teal and ALK2WT-Phosp is shown in orange, taken over trajectory subsets indicated in the previous wavelet analysis figure. (TIF) [file pcbi.1005711.s004.tif]

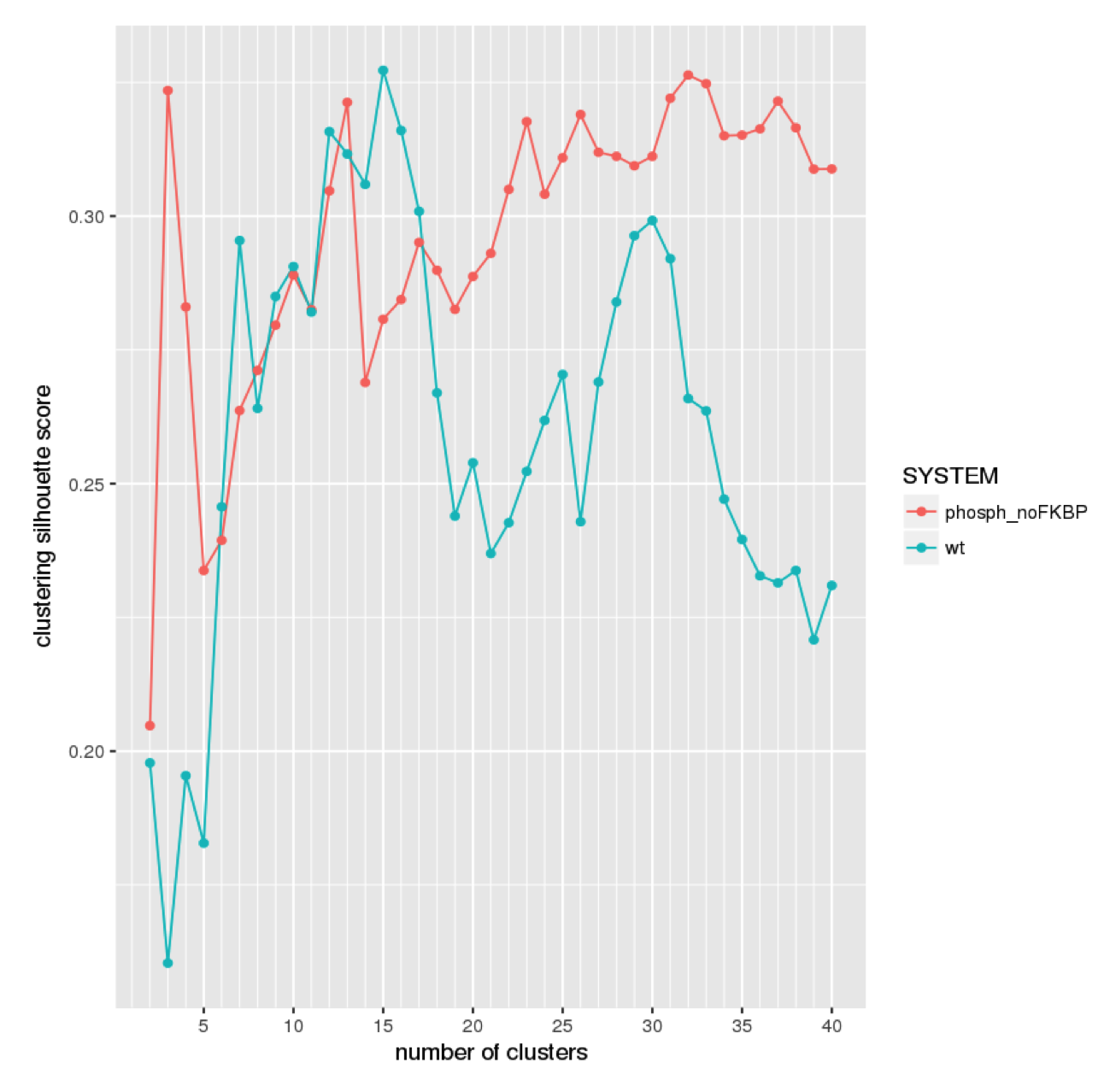

Supplement: S5 Fig — This figure was generated by computing silhouette scores for consecutive k-cluster cuts taken from the hierarchical clustering of the frame-wise WAFEX data. Hierarchical clustering was computed using Euclidean distance norms over the log of the wavelet intensities at each frame along with Ward similarity scoring as implemented in the hclust function of the fastcluster package in R. (TIF) [file pcbi.1005711.s005.tif]

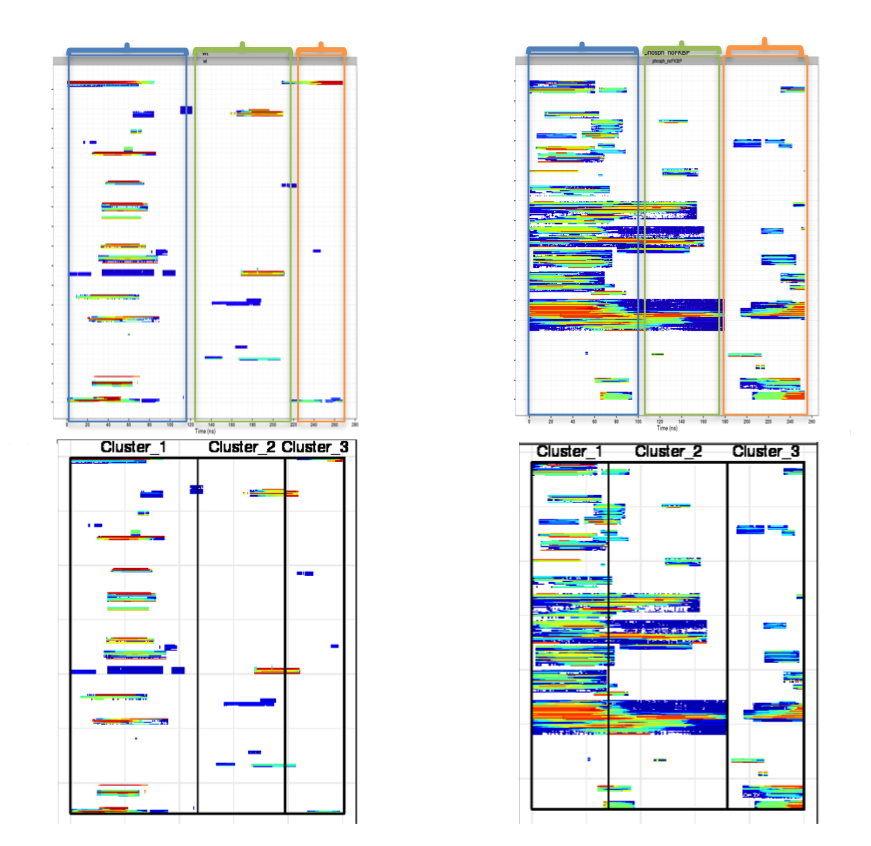

Supplement: S6 Fig — Top: Clustering performed by eye. Bottom: Automated Hierarchical Clustering cut to 3 clusters. Left: Unphosphorylated ALK2 system with FKBP12 bound. Right: Phosphorylated ALK2 system without FKBP12 bound. (TIF) [file pcbi.1005711.s006.tif]

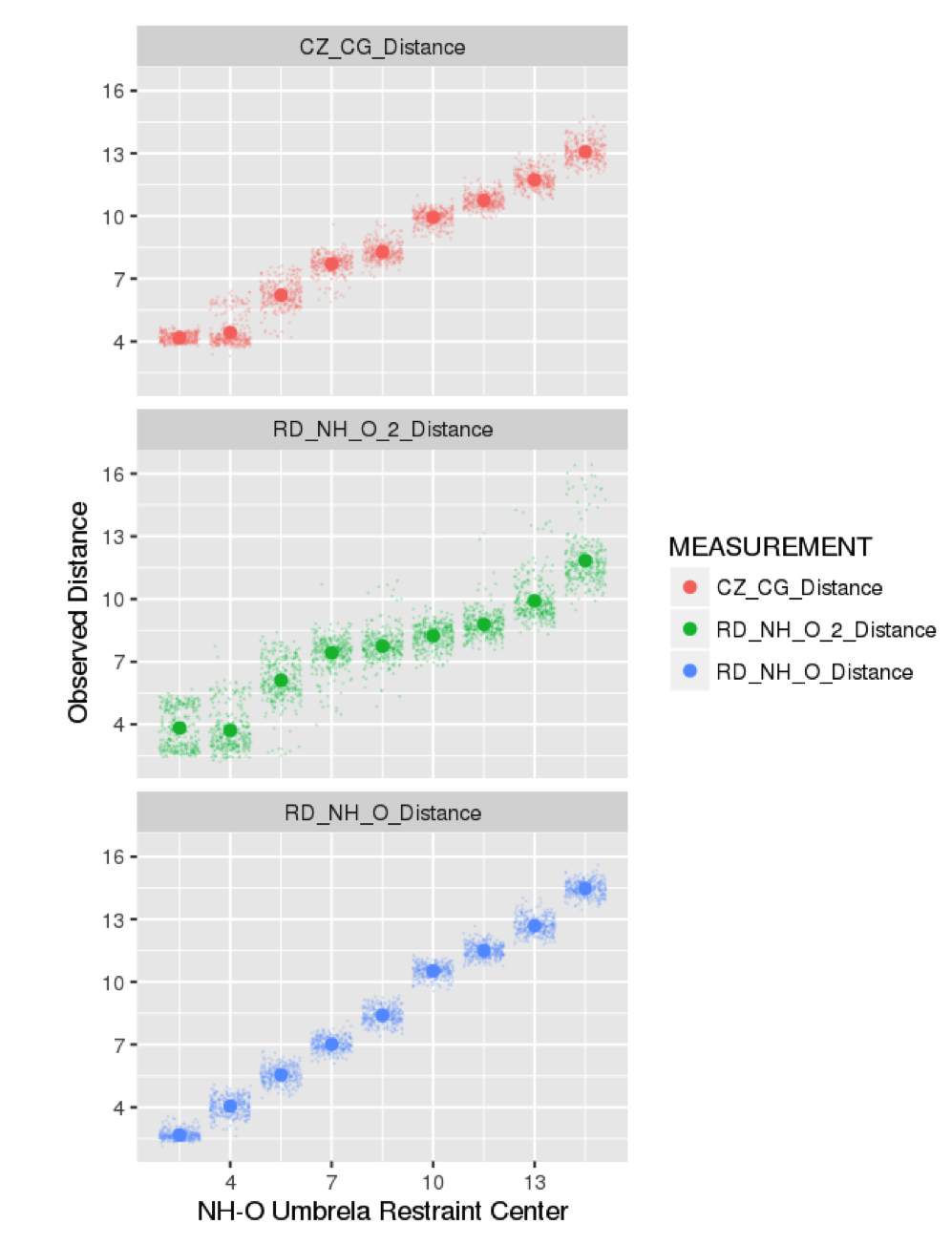

Supplement: S7 Fig — Top: Correlation plot for distance between R375(Cζ)-D354(Cγ) and umbrella restraint center. Middle: Correlation plot for distance between center of mass of hydrogens of non-restrained NH2 moiety of R375 guanidine group to non-restrained oxygen of D354 side-chain carboxylic acid group. Bottom: Correlation plot for distance between center of mass of hydrogens of restrained NH2 moiety of R375 guanidine group and restrained oxygen of D354 side-chain carboxylic acid group, i.e. umbrella restraint center. (TIF) [file pcbi.1005711.s007.tif]
